# (*R*)-1-Phenyl­ethyl­ammonium trifluoro­acetate

**DOI:** 10.1107/S1600536810013565

**Published:** 2010-04-21

**Authors:** María-Guadalupe Hernández Linares, Gabriel Guerrero Luna, Sylvain Bernès

**Affiliations:** a Escuela de Ingeniería Química, Universidad del Istmo, Ciudad Universitaria s/n, 70760 Sto. Domingo Tehuantepec, Oax., Mexico; b DEP Facultad de Ciencias Químicas, UANL, Guerrero y Progreso S/N, Col. Treviño, 64570 Monterrey, NL, Mexico

## Abstract

In the crystal structure of the title salt, C_8_H_12_N^+^·C_2_F_3_O_2_^−^, all of the ammonium H atoms serve as donors for hydrogen bonds to carboxyl­ate O atoms, forming an *R*_4_^3^(10) ring motif based on two cations and two anions. Since both cations and anions act as inter-ion bridging groups, *R*(10) rings aggregate in a one-dimensional supra­molecular network by sharing the strongest N—H⋯O bond. Edge-sharing motifs lie on the twofold screw axis parallel to [010], and anti­parallel packing of these 2_1_-column structural units results in the crystal structure. This arrangement is one of the most commonly occurring in conglomerates of chiral 1-phenyl­ethyl­amine with achiral monocarboxylic acids, confirming that these ionic salts are particularly robust supra­molecular heterosynthons useful in crystal engineering.

## Related literature

For graph-set analysis, see: Etter (1990 ▶); Bernstein *et al.* (1995 ▶). For characteristic structural patterns found in crystal salts of 1-phenyl­ethyl­amine and monocarboxylic acids, see: Kinbara, Hashimoto *et al.* (1996 ▶); Kinbara, Kai *et al.* (1996 ▶); Lemmerer *et al.* (2008 ▶). For related chiral salt structures, see: Johansen *et al.* (1998 ▶); Boussac *et al.* (2002 ▶); Lemmerer *et al.* (2008 ▶).
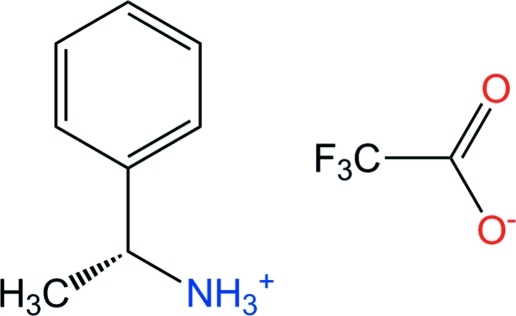


## Experimental

### 

#### Crystal data

C_8_H_12_N^+^·C_2_F_3_O_2_^−^
*M* *_r_* = 235.21
Orthorhombic, 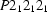

*a* = 6.7821 (5) Å
*b* = 6.9887 (8) Å
*c* = 24.378 (2) Å
*V* = 1155.49 (19) Å^3^
*Z* = 4
Mo *K*α radiation
μ = 0.13 mm^−1^
*T* = 298 K
0.60 × 0.44 × 0.40 mm

#### Data collection

Siemens P4 diffractometer
3079 measured reflections
1808 independent reflections
1288 reflections with *I* > 2σ(*I*)
*R*_int_ = 0.020
3 standard reflections every 97 reflections  intensity decay: 1%

#### Refinement

*R*[*F* ^2^ > 2σ(*F* ^2^)] = 0.041
*wR*(*F* ^2^) = 0.112
*S* = 1.03
1808 reflections
156 parameters
H atoms treated by a mixture of independent and constrained refinement
Δρ_max_ = 0.16 e Å^−3^
Δρ_min_ = −0.13 e Å^−3^

### 

Data collection: *XSCANS* (Siemens, 1996 ▶); cell refinement: *XSCANS*; data reduction: *XSCANS* (Siemens, 1996 ▶); program(s) used to solve structure: *SHELXTL-Plus* (Sheldrick, 2008 ▶); program(s) used to refine structure: *SHELXTL-Plus*; molecular graphics: *Mercury* (Macrae *et al.*, 2008 ▶); software used to prepare material for publication: *SHELXTL-Plus*.

## Supplementary Material

Crystal structure: contains datablocks I, global. DOI: 10.1107/S1600536810013565/pb2025sup1.cif

Structure factors: contains datablocks I. DOI: 10.1107/S1600536810013565/pb2025Isup2.hkl

Additional supplementary materials:  crystallographic information; 3D view; checkCIF report

## Figures and Tables

**Table 1:** Hydrogen-bond geometry (Å, °)

*D*—H⋯*A*	*D*—H	H⋯*A*	*D*⋯*A*	*D*—H⋯*A*
N1—H1*A*⋯O1	0.90 (3)	1.92 (3)	2.812 (3)	171 (3)
N1—H1*B*⋯O2^i^	0.92 (3)	1.97 (3)	2.818 (3)	154 (3)
N1—H1*C*⋯O2^ii^	0.90 (3)	1.92 (3)	2.816 (2)	175 (3)

Symmetry codes: (i) 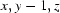; (ii) 
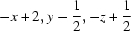
.

**Fig. 1.**
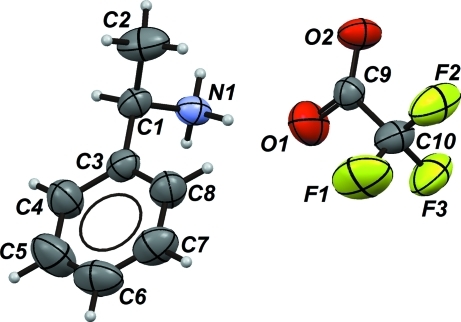
The structure of the title compound, with displacement ellipsoids at the 50% probability level for non-H atoms.

**Fig. 2.**
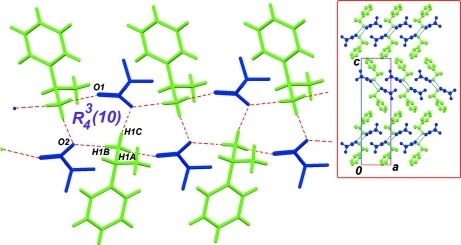
The hydrogen-bonding network in the title compound (hydrogen bonds are dashed). The inset represent the packing structure viewed down the axis of the 21–column (b axis). H atoms have been omitted for clarity. In both figures, cations are green and anions blue.

## supplementary crystallographic information

### Comment

In their works about optical resolution of conglomerates, Kinbara *et al.* noted that characteristic hydrogen-bond networks were formed in the salt crystals of 1-phenylethylamine and 1-(4-*iso*propylphenyl)ethylamine with cinnamic acid (Kinbara, Kai *et al.*, 1996). They suggested that "the pattern of hydrogen bonds plays a significant role in the formation of conglomerates" (Kinbara, Hashimoto *et al.*, 1996). In the specific case of salts of chiral 1-phenylethylamine with achiral monocarboxylic acids, a number of structural determinations indeed showed that two predominant supramolecular arrangements are favored by the charge assisted N—H···O hydrogen bonds, which result in crystals belonging to *P*2_1_ or *P*2_1_2_1_2_1_ space groups (Lemmerer *et al.*, 2008): cations and anions associate through quite strong hydrogen bonds to form *C*_2_^1^(4)*C*_2_^2^(6)[*R*_4_^3^(10)] motifs (Etter, 1990; Bernstein *et al.*, 1995). This basic unit has hydrogen bonds with translational units, forming an infinite columnar structure, which generates a screw axis in the crystal structure (invariably a 2_1_ axis). This supramolecular structure, referred as '2_1_–column' in the Kinbara's reports, may be arranged in a parallel packing in the crystal, which then belongs to *P*2_1_ space group, or in an antiparallel fashion, generating *P*2_1_2_1_2_1_ crystals.

The chiral title salt (Fig. 1) clearly falls in the latter category. Both the cation and anion are placed in general positions in an orthorhombic unit cell. All ammonium H atoms form hydrogen bonds with carboxylate O atoms, giving a ring motif *R*_4_^3^(10), as shown in Fig. 2. The strongest hydrogen bond, N1—H1C···O2*^ii^* is common to two rings motifs. The repetition of the motif in the [010] direction generates homochiral (*R*)–2_1_–columns. This 1D supramolecular network includes larger ring motifs, which appear if shared contacts are omitted. The sequence of sub-rings nest is *R*_4_^3^(10) →*R*_6_^5^(16) →*R*_8_^7^(22) →*R*_10_^9^(28) → ··· →*R*_2_*_n_*^2^*^n^*^-1^(6*n*-2) [with *n* > 1]. The shortest contact between neighboring 2_1_–columns is N1—H1B···F2*^i^*, which should be regarded as a van der Waals contact rather than as an actual hydrogen bond. As a consequence, an antiparallel arrangement of 2_1_–columns is favored (Fig. 2, inset), which is, in turn, reflected in the *P*2_1_2_1_2_1_ space group. Such crystal structures were obtained for numerous 1-phenylethylamine salts including different anions, *e.g*. bromofluoroacetate (Boussac *et al.*, 2002), *m*-iodobenzoate (Lemmerer *et al.*, 2008) or more complex, bulky carboxylate derivatives (Johansen *et al.*, 1998).

The above description is thus in line with expectations from previous reported structures, and confirms that salts based on chiral 1-phenylethylamine and achiral monocarboxylic acids are robust heterosynthons, useful for crystal engineering and crystal structure prediction. The feature should however not been transferred to other salts (or worse, to cocrystals) of 1-phenylethylamine, which stabilize different supramolecular motifs, if any.

### Experimental

The title salt crystallized when attempting to synthesize a diimine organic ligand. A mixture of (*S*)-6-acetyloxy-5-methyl-2,3-hexanedione (1 g, 5.37 mmol) and Na_2_SO_4_ (4 g) in chloroform (10 ml) was stirred for 5 min. A catalytic amount of trifluoroacetic acid and 2 equiv. of (*R*)-(+)-α-phenylethylamine (10.6 mmol) were added and the mixture was refluxed (*ca*. 353 K) under inert atmosphere, until starting materials were not detected by TLC (*ca*. 2 h). After evaporation under reduced pressure, the crude was recrystallized from CH_2_Cl_2_ at 298 K, affording, among other products, the title salt.

### Refinement

As no heavy atoms are present in the crystal and data were measured at room-temperature using Mo *K*α radiation, no absorption correction was applied to the raw data. Because of insufficient anomalous scattering effects, the Flack parameter could not be reliably determined, and measured Friedel pairs (796) were merged. Absolute configuration was assigned by reference to the chiral amine used as starting material, assuming that no inversion occurred during crystallization. Ammonium H atoms were refined with free coordinates, in order to get accurate dimensions for hydrogen bonds. Other H atoms were placed in idealized positions and refined as riding to their carrier atoms, with bond lengths fixed to 0.93 (aromatic CH), 0.96 (methyl CH_3_) or 0.98 Å (methine CH). Isotropic displacement parameters for H atoms were calculated as *U*_iso_(H) = 1.2*U*_eq_(C) for aromatic CH groups and *U*_iso_(H) = 1.5*U*_eq_(C, N) for other groups. The methyl group was considered as a rigid group free to rotate about its C—C bond.

### Crystal data

**Table:**

C_8_H_12_N^+^·C_2_F_3_O_2_^−^	*F*(000) = 488
*M**_r_* = 235.21	*D*_x_ = 1.352 Mg m^−^^3^
Orthorhombic, *P*2_1_2_1_2_1_	Mo *K*α radiation, λ = 0.71073 Å
Hall symbol: P 2ac 2ab	Cell parameters from 70 reflections
*a* = 6.7821 (5) Å	θ = 4.5–12.5°
*b* = 6.9887 (8) Å	µ = 0.13 mm^−^^1^
*c* = 24.378 (2) Å	*T* = 298 K
*V* = 1155.49 (19) Å^3^	Prism, colorless
*Z* = 4	0.60 × 0.44 × 0.40 mm

### Data collection

**Table:**

Siemens P4 diffractometer	*R*_int_ = 0.020
Radiation source: fine-focus sealed tube	θ_max_ = 29.0°, θ_min_ = 1.7°
graphite	*h* = −9→4
2θ/ω scans	*k* = −9→1
3079 measured reflections	*l* = −33→1
1808 independent reflections	3 standard reflections every 97 reflections
1288 reflections with *I* > 2σ(*I*)	intensity decay: 1%

### Refinement

**Table:**

Refinement on *F*^2^	Secondary atom site location: difference Fourier map
Least-squares matrix: full	Hydrogen site location: inferred from neighbouring sites
*R*[*F*^2^ > 2σ(*F*^2^)] = 0.041	H atoms treated by a mixture of independent and constrained refinement
*wR*(*F*^2^) = 0.112	*w* = 1/[σ^2^(*F*_o_^2^) + (0.0464*P*)^2^ + 0.1579*P*] where *P* = (*F*_o_^2^ + 2*F*_c_^2^)/3
*S* = 1.03	(Δ/σ)_max_ < 0.001
1808 reflections	Δρ_max_ = 0.16 e Å^−^^3^
156 parameters	Δρ_min_ = −0.13 e Å^−^^3^
0 restraints	Extinction correction: *SHELXTL-Plus* (Sheldrick, 2008), Fc^*^=kFc[1+0.001xFc^2^λ^3^/sin(2θ)]^-1/4^
0 constraints	Extinction coefficient: 0.045 (4)
Primary atom site location: structure-invariant direct methods	

### Fractional atomic coordinates and isotropic or equivalent isotropic displacement parameters (Å^2^)

**Table:**

	*x*	*y*	*z*	*U*_iso_*/*U*_eq_	
N1	0.9254 (3)	0.1712 (3)	0.19995 (7)	0.0480 (4)	
H1C	1.002 (5)	0.206 (4)	0.2286 (11)	0.072*	
H1B	0.853 (5)	0.062 (5)	0.2039 (11)	0.072*	
H1A	0.840 (5)	0.268 (5)	0.1966 (11)	0.072*	
C1	1.0532 (3)	0.1429 (4)	0.15044 (8)	0.0513 (5)	
H1	1.1377	0.0316	0.1571	0.062*	
C2	1.1846 (5)	0.3153 (5)	0.14290 (11)	0.0818 (9)	
H2C	1.2617	0.3000	0.1102	0.123*	
H2B	1.2709	0.3273	0.1739	0.123*	
H2A	1.1049	0.4283	0.1398	0.123*	
C3	0.9260 (3)	0.1001 (4)	0.10055 (8)	0.0502 (5)	
C4	0.9482 (5)	−0.0721 (4)	0.07311 (11)	0.0706 (7)	
H4	1.0390	−0.1616	0.0857	0.085*	
C5	0.8359 (6)	−0.1118 (5)	0.02695 (12)	0.0893 (10)	
H5	0.8526	−0.2272	0.0086	0.107*	
C6	0.7020 (5)	0.0165 (5)	0.00854 (11)	0.0819 (10)	
H6	0.6265	−0.0114	−0.0223	0.098*	
C7	0.6776 (4)	0.1863 (5)	0.03497 (10)	0.0743 (8)	
H7	0.5859	0.2744	0.0221	0.089*	
C8	0.7891 (4)	0.2282 (4)	0.08117 (9)	0.0633 (7)	
H8	0.7710	0.3442	0.0992	0.076*	
C9	0.6937 (4)	0.6770 (3)	0.19540 (9)	0.0482 (5)	
O1	0.6911 (4)	0.5019 (3)	0.19191 (10)	0.0921 (7)	
O2	0.8190 (3)	0.7849 (3)	0.21505 (7)	0.0620 (5)	
C10	0.5086 (4)	0.7725 (4)	0.17224 (11)	0.0613 (6)	
F1	0.4723 (3)	0.7214 (4)	0.12145 (7)	0.1175 (8)	
F2	0.5178 (3)	0.9610 (2)	0.17221 (11)	0.1065 (8)	
F3	0.3482 (2)	0.7254 (3)	0.20053 (8)	0.0914 (6)	

### Atomic displacement parameters (Å^2^)

**Table:**

	*U*^11^	*U*^22^	*U*^33^	*U*^12^	*U*^13^	*U*^23^
N1	0.0484 (10)	0.0459 (9)	0.0498 (9)	−0.0030 (9)	−0.0118 (9)	0.0002 (9)
C1	0.0420 (10)	0.0574 (13)	0.0545 (11)	0.0045 (11)	−0.0077 (10)	−0.0010 (10)
C2	0.0655 (16)	0.105 (2)	0.0744 (16)	−0.029 (2)	−0.0104 (14)	0.0154 (17)
C3	0.0429 (11)	0.0622 (13)	0.0455 (10)	0.0012 (12)	−0.0017 (9)	0.0003 (10)
C4	0.0760 (17)	0.0669 (16)	0.0689 (14)	0.0113 (16)	−0.0136 (15)	−0.0104 (13)
C5	0.116 (3)	0.082 (2)	0.0699 (15)	−0.003 (2)	−0.0199 (18)	−0.0186 (16)
C6	0.085 (2)	0.106 (3)	0.0544 (13)	−0.022 (2)	−0.0162 (15)	−0.0043 (16)
C7	0.0650 (16)	0.099 (2)	0.0585 (13)	0.0027 (18)	−0.0157 (13)	0.0114 (15)
C8	0.0618 (14)	0.0706 (16)	0.0576 (12)	0.0107 (14)	−0.0108 (12)	−0.0038 (12)
C9	0.0487 (12)	0.0465 (12)	0.0495 (10)	0.0049 (10)	−0.0017 (10)	−0.0005 (9)
O1	0.0946 (16)	0.0488 (11)	0.1330 (18)	0.0160 (11)	−0.0199 (16)	−0.0080 (11)
O2	0.0575 (10)	0.0609 (10)	0.0677 (9)	−0.0061 (9)	−0.0241 (8)	0.0114 (8)
C10	0.0477 (13)	0.0611 (14)	0.0753 (14)	−0.0048 (13)	−0.0089 (12)	0.0110 (13)
F1	0.1021 (14)	0.173 (2)	0.0768 (10)	−0.0089 (18)	−0.0389 (10)	0.0115 (13)
F2	0.0614 (10)	0.0583 (10)	0.200 (2)	0.0064 (9)	−0.0258 (13)	0.0349 (11)
F3	0.0526 (8)	0.0911 (13)	0.1303 (14)	−0.0024 (10)	0.0155 (9)	0.0106 (12)

### Geometric parameters (Å, °)

**Table:**

N1—C1	1.499 (3)	C5—C6	1.352 (5)
N1—H1C	0.90 (3)	C5—H5	0.9300
N1—H1B	0.92 (3)	C6—C7	1.361 (4)
N1—H1A	0.90 (3)	C6—H6	0.9300
C1—C2	1.510 (4)	C7—C8	1.388 (3)
C1—C3	1.521 (3)	C7—H7	0.9300
C1—H1	0.9800	C8—H8	0.9300
C2—H2C	0.9600	C9—O1	1.227 (3)
C2—H2B	0.9600	C9—O2	1.233 (3)
C2—H2A	0.9600	C9—C10	1.530 (3)
C3—C8	1.374 (3)	C10—F1	1.312 (3)
C3—C4	1.385 (3)	C10—F2	1.319 (3)
C4—C5	1.387 (4)	C10—F3	1.329 (3)
C4—H4	0.9300		
			
C1—N1—H1C	109.1 (18)	C5—C4—H4	119.8
C1—N1—H1B	106.6 (18)	C6—C5—C4	120.4 (3)
H1C—N1—H1B	117 (2)	C6—C5—H5	119.8
C1—N1—H1A	113.5 (18)	C4—C5—H5	119.8
H1C—N1—H1A	104 (2)	C5—C6—C7	120.2 (3)
H1B—N1—H1A	107 (2)	C5—C6—H6	119.9
N1—C1—C2	109.5 (2)	C7—C6—H6	119.9
N1—C1—C3	109.99 (17)	C6—C7—C8	120.1 (3)
C2—C1—C3	113.23 (19)	C6—C7—H7	119.9
N1—C1—H1	108.0	C8—C7—H7	119.9
C2—C1—H1	108.0	C3—C8—C7	120.7 (3)
C3—C1—H1	108.0	C3—C8—H8	119.7
C1—C2—H2C	109.5	C7—C8—H8	119.7
C1—C2—H2B	109.5	O1—C9—O2	130.3 (3)
H2C—C2—H2B	109.5	O1—C9—C10	113.4 (2)
C1—C2—H2A	109.5	O2—C9—C10	116.2 (2)
H2C—C2—H2A	109.5	F1—C10—F2	106.3 (2)
H2B—C2—H2A	109.5	F1—C10—F3	105.6 (2)
C8—C3—C4	118.3 (2)	F2—C10—F3	106.6 (3)
C8—C3—C1	122.0 (2)	F1—C10—C9	112.6 (2)
C4—C3—C1	119.7 (2)	F2—C10—C9	113.4 (2)
C3—C4—C5	120.4 (3)	F3—C10—C9	111.85 (19)
C3—C4—H4	119.8		
			
N1—C1—C3—C8	61.4 (3)	C4—C3—C8—C7	−0.6 (4)
C2—C1—C3—C8	−61.5 (3)	C1—C3—C8—C7	178.9 (2)
N1—C1—C3—C4	−119.1 (2)	C6—C7—C8—C3	0.4 (4)
C2—C1—C3—C4	118.0 (3)	O1—C9—C10—F1	−54.5 (3)
C8—C3—C4—C5	0.7 (4)	O2—C9—C10—F1	126.5 (2)
C1—C3—C4—C5	−178.8 (3)	O1—C9—C10—F2	−175.2 (3)
C3—C4—C5—C6	−0.7 (5)	O2—C9—C10—F2	5.8 (3)
C4—C5—C6—C7	0.5 (5)	O1—C9—C10—F3	64.2 (3)
C5—C6—C7—C8	−0.4 (5)	O2—C9—C10—F3	−114.8 (3)

### Hydrogen-bond geometry (Å, °)

**Table:**

*D*—H···*A*	*D*—H	H···*A*	*D*···*A*	*D*—H···*A*
N1—H1A···O1	0.90 (3)	1.92 (3)	2.812 (3)	171 (3)
N1—H1B···O2^i^	0.92 (3)	1.97 (3)	2.818 (3)	154 (3)
N1—H1C···O2^ii^	0.90 (3)	1.92 (3)	2.816 (2)	175 (3)
N1—H1B···F2^i^	0.92 (3)	2.50 (3)	3.202 (3)	134 (2)

Symmetry codes: (i) *x*, *y*−1, *z*; (ii) −*x*+2, *y*−1/2, −*z*+1/2.
